# Photodynamic and Antibiotic Therapy Impair the Pathogenesis of *Enterococcus faecium* in a Whole Animal Insect Model

**DOI:** 10.1371/journal.pone.0055926

**Published:** 2013-02-14

**Authors:** José Chibebe Junior, Beth B. Fuchs, Caetano P. Sabino, Juliana C. Junqueira, Antonio O. C. Jorge, Martha S. Ribeiro, Michael S. Gilmore, Louis B. Rice, George P. Tegos, Michael R. Hamblin, Eleftherios Mylonakis

**Affiliations:** 1 Department of Biosciences and Oral Diagnosis, Univ Estadual Paulista/UNESP, São José dos Campos, São Paulo, Brazil; 2 Division of Infectious Diseases, Massachusetts General Hospital, Boston, Massachusetts, United States of America; 3 Department of Restorative Dentistry, Faculty of Pindamonhangaba, Pindamonhangaba, São Paulo, Brazil; 4 Wellman Center for Photomedicine, Massachusetts General Hospital, Boston, Massachusetts, United States of America; 5 Center for Lasers and Applications, Nuclear and Energy Research Institute, São Paulo, São Paulo, Brazil; 6 Massachusetts Eye and Ear Infirmary, Harvard Medical School, Boston, Massachusetts, United States of America; 7 Warren Alpert Medical School, Brown University/Rhode Island and Miriam Hospitals, Providence, Rhode Island, United States of America; 8 Department of Dermatology, Harvard Medical School, Boston, Massachusetts, United States of America; 9 Department of Pathology and Center for Molecular Discovery, University of New Mexico, Albuquerque, New Mexico, United States of America; 10 Division of Health Sciences and Technology, Harvard-Massachusetts Institute of Technology, Cambridge, Massachusetts, United States of America; Institut Pasteur, France

## Abstract

*Enterococcus faecium* has emerged as one of the most important pathogens in healthcare-associated infections worldwide due to its intrinsic and acquired resistance to many antibiotics, including vancomycin. Antimicrobial photodynamic therapy (aPDT) is an alternative therapeutic platform that is currently under investigation for the control and treatment of infections. PDT is based on the use of photoactive dye molecules, widely known as photosensitizer (PS). PS, upon irradiation with visible light, produces reactive oxygen species that can destroy lipids and proteins causing cell death. We employed *Galleria mellonella* (the greater wax moth) caterpillar fatally infected with *E. faecium* to develop an invertebrate host model system that can be used to study the antimicrobial PDT (alone or combined with antibiotics). In the establishment of infection by *E. faecium* in *G. mellonella*, we found that the *G. mellonella* death rate was dependent on the number of bacterial cells injected into the insect hemocoel and all *E. faecium* strains tested were capable of infecting and killing *G. mellonella*. Antibiotic treatment with ampicillin, gentamicin or the combination of ampicillin and gentamicin prolonged caterpillar survival infected by *E. faecium* (*P* = 0.0003, *P* = 0.0001 and *P* = 0.0001, respectively). In the study of antimicrobial PDT, we verified that methylene blue (MB) injected into the insect followed by whole body illumination prolonged the caterpillar survival (*P* = 0.0192). Interestingly, combination therapy of larvae infected with vancomycin-resistant *E. faecium*, with antimicrobial PDT followed by vancomycin, significantly prolonged the survival of the caterpillars when compared to either antimicrobial PDT (*P* = 0.0095) or vancomycin treatment alone (*P* = 0.0025), suggesting that the aPDT made the vancomycin resistant *E. faecium* strain more susceptible to vancomycin action. In summary, *G. mellonella* provides an invertebrate model host to study the antimicrobial PDT and to explore combinatorial aPDT-based treatments.

## Introduction

Enterococci are part of the gastrointestinal tract of humans [1]–[3], but due to intrinsic and acquired resistance to many antibiotics, they have become leading causes of nosocomial infections worldwide [4]–[7]. *Enterococcus faecalis* and *Enterococcus faecium* account for 95% of clinical isolates from the genus *Enterococcus*, and are isolated from patients with endocarditis, bloodstream infection, wound and surgical-site infection, and intra-abdominal and urinary tract infection [3], [8], [9]. In dentistry, they are frequently associated with chronic periodontitis and persistent endodontic infections [10]–[12]. In the 1980s and early 1990s, more than 90% of all enterococcal infections were caused by *E. faecalis* and only 5–10% by *E. faecium*. Due to the acquisition of the virulence determinants as well as acquired antibiotic resistance, this ratio has changed, and currently, *E. faecium* is associated with between 38–75% of all enterococcal infections [4], [13].

The increased resistance of bacteria to antibiotics has emerged as one of the most important clinical challenges of this century, highlighting the need for new and effective antimicrobial countermeasures against resistant bacteria and especially the “ESKAPE” pathogens (*Enterococcus faecium*, *Staphylococcus aureus*, *Klebsiella pneumoniae*, *Acinetobacter baumanii*, *Pseudomonas aeruginosa* and *Enterobacter* spp.) [14], [15]. Photodynamic therapy (PDT), is a light-based technology platform [16] that uses harmless visible light in combination with non-toxic dye, called photosensitizer (PS), to control infections. PSs are usually organic aromatic molecules with a high degree of electron delocalization [17]. Porphyrins, chlorins, bacteriochlorins, phthalocyanines as well as a plethora of dyes with different molecular frameworks have been proposed as antimicrobial PSs [18]–[20]. Historically PDT has had a prominent role in cancer therapy and is also currently used to treat age-related macular degeneration [21]. Currently, PDT is being investigated as an alternative treatment for localized infections [22]. Dental, dermatologic as well as oral soft tissue infections are areas of special interest for antimicrobial PDT (aPDT) research [14], [23]–[27].

The use of mammalian models for studying pathogenesis and the efficacy of antimicrobial treatments *in vivo* is costly and cumbersome [28]. The use of invertebrate model hosts has important advantages for obtaining *in vivo* data at low cost and with no special housing requirements or need for regulatory approval. The larvae of the greater wax moth, *Galleria mellonella*, has been used to study host-pathogen interaction as an alternative to mammalian models and a positive correlation between microbial virulence in mammalian hosts and in *G. mellonella* has been demonstrated for a range of organisms [29]–[31]. Advantages of the *Galleria* model include facile inoculation of microorganisms and the ability to thrive at 37°C.

*G. mellonella* is an ideal model to examine aPDT *in vivo*: the photosensitizer can be injected into the insect haemocoel and the relatively translucent body facilitates light delivery activating the PS. Because of the importance of *E. faecium* as a hospital pathogen that is often resistant to most antimicrobial therapies, it was of interest to examine the utility of aPDT in limiting this infection. We characterized the *G. mellonella* model for *E. faecium* infection and tested methylene blue (MB) mediated aPDT and aPDT-antibiotic combination therapy for efficacy.

## Materials and Methods

### Microbial Strains and Culture Conditions

The strains of *E. faecium* used in these experiments are summarized in **Table 1**. We tested strains of *E. faecium* with different phenotypic characteristics. We also compared efficacy of aPDT for treating infection caused by *E. faecalis*.

**Table 1:** Bacterial strains used in this study.

Strain	Relevant characteristics	Reference
***E. faecium***		
E007	clinical isolate; pMV158GFP; tetracycline resistance	[64]
1,231,410	clinical isolate; vancomycin resistance	[40]
D344R	clinical isolate; ampicillin resistance	[65]
2158	TX1330RF/(pHylEfmTX16); virulent in mouse peritonitis model	[32]
***E. faecalis***		
OG1RF	rifampin and fusidic acid resistance	[35]
V583	blood culture isolated; vancomycin resistance	[36]

*E. faecium* and *E. faecalis* inocula were prepared by growing bacteria aerobically in brain-heart-infusion (BHI) at 37°C without shaking (overnight growth). The culture concentration was determined by optical density and compared to a standard curve determined by plating serial dilutions on BHI agar. Cell numbers were assessed at 24 h and expressed in colony forming units (CFU) per ml. Prior to injection, cells were washed twice in phosphate-buffered saline (PBS) and diluted in PBS to the desired concentration.

### *G. mellonella* Injection

*G. mellonella* in the final instar larval stage (250–350 mg body weight) were stored in the dark at 15°C and used within 7 days from shipment (Vanderhorst Wholesale, St. Marys, OH). Two control groups were included in each experiment: one inoculated with PBS as a control for physical trauma, and the other not injected as a control for general viability. A 10 µl Hamilton syringe was used to inject 10 µl inoculum aliquots into the hemocoel of each larvae via the last left proleg. After injection, larvae were incubated at 37°C in plastic containers.

### *G. mellonella* Survival Assays

After injection, larvae were observed every 24 h, and considered dead when they displayed no movement in response to touch. Sixteen randomly chosen *G. mellonella* larvae were used per group in all assays. Survival curves were constructed by the Kaplan-Meier method and compared by the Log-rank (Mantel-Cox) test using Graph Pad Prism statistical software. A *P* value <0.05 was considered statistically significant. All experiments were repeated at least twice, and representative experiments are presented.

### Persistence of *E. faecium* in the Hemolymph

The number of bacterial cells in the hemolymph was measured at 0, 2, 4, 8, 12 and 24 h after larvae were infected with the *E. faecium* strain E007. At each indicated time-point, 5 surviving larvae per group were bled by insertion of a lancet into the hemocoel. Hemolymph from 5 larvae was pooled into 1.5 ml Eppendorf tubes in a final volume of approximately 130 µL. Then, the hemolymph was homogenized, serially diluted, and plated on BHI agar containing tetracycline (12.5 mg/L), kanamycin (45 mg/L) and amphotericin B (3 mg/L), to prevent contamination by other bacteria or fungal cells. Plates were incubated aerobically at 37°C for 24 h, and colonies were counted in each pool (CFU/pool).

### Administration of Antibacterial Agents

Antibiotics were injected within 120 min after the infection of larvae with a lethal dose of *E. faecium*. The antibiotics and doses included ampicillin (150 mg/kg), streptomycin (15 mg/kg), gentamicin (6 mg/kg) and vancomycin (50 mg/kg). A different proleg was used for the infection and antibiotic injection. As control group, the caterpillars received PBS injections. After that, killing curves were plotted using the Log-rank (Mantel-Cox) test.

### Photodynamic Therapy

The phenothiazinium salt methylene blue (MB, Sigma Aldrich) was used as the PS in this study. MB solutions at a final working concentration of 1 mM were prepared by dissolving the dye in distilled and deionized filter sterilized water (ddH_2_O). A new PS solution was prepared on the same day of each experiment. After the PS injection, larvae were maintained in the dark until the time of light irradiation.

A broad-band non coherent light source (LumaCare, Newport Beach, CA) was used for light delivery. This device was fitted with a 660±15 nm band-pass filter probe that was employed to produce a uniform spot for illumination. The optical power was measured using a power meter (PM100D power/energy meter, Thorlabs, Inc., Newton, NJ).

All experiments were performed as follows: *G. mellonella* received the PS injection (10 µL) 90 min after the bacterial infection. We waited for at least 30 additional min after the PS injection to allow a good dispersion of the PS into the insect body, prior to the light irradiation. After the irradiation, survival curves were plotted using the log-rank (Mantel-Cox) test.

## Results

**Figure 1:**
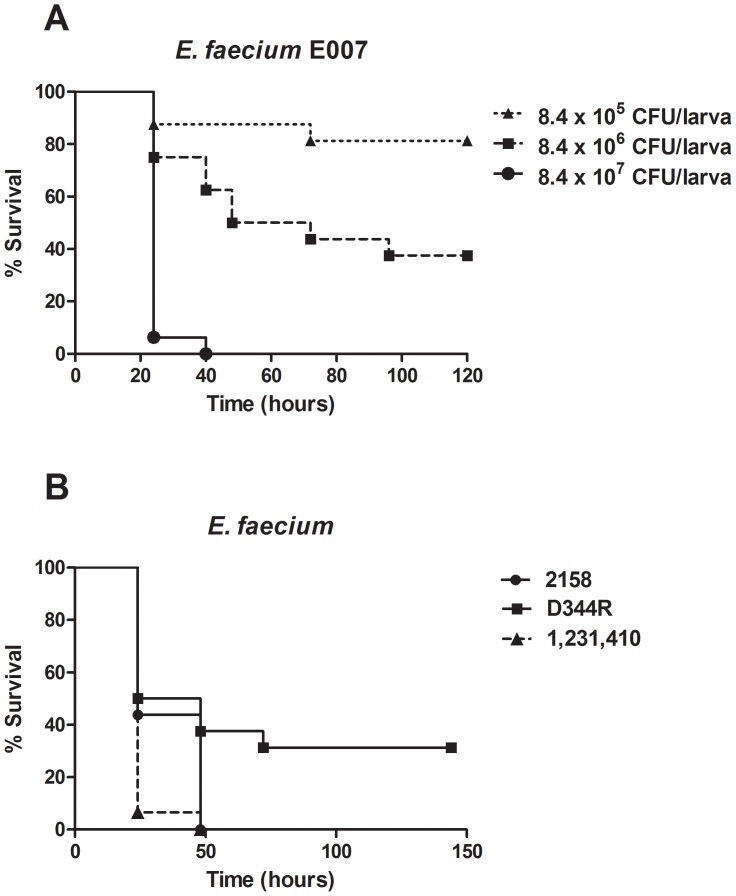
Killing of *G. mellonella* larvae by *E. faecium*. Comparison of survival curves by Log-rank test: A) *G. mellonella* survival after injection of different inocula of *E. faecium* (10^5^, 10^6^ or 10^7^ CFU/larva) and maintained at 37°C. Injection with 8.4×10^7^ CFU/larva resulted in significantly higher death rate, compared to injection with 8.4×10^6^ CFU/larva (*P* = 0.0001) or 8.4×10^5^ CFU/larva (*P* = 0.0001). Injection with 8.4×10^6^ CFU/larva resulted in significantly higher death rate compared to injection with 8.4×10^5^ CFU/larva (*P* = 0.0139). B) Killing of *G. mellonella* by *E. faecium* D344R ampicillin resistant (3.0×10^7^ CFU/larva), *E. faecium* 1,231,410 vancomycin resistant (4.8×10^7^ CFU/larva) and *E. faecium* 2158 that was tested previously in mouse peritonitis model (1.25×10^7^ CFU/larva). A representative example was used for each group.

Initially, a set of experiments was performed to provide a comprehensive understanding of the host response following *E. faecium* infection. We injected different inocula of *E. faecium* E007 (clinical isolate tetracycline resistant) in *G. mellonella* and it was observed that the increasing concentrations (10^5^, 10^6^ and 10^7 ^CFU/larva) of the *E. faecium* cell numbers resulted in progressively decreasing survival of the infected larvae (Fig. 1A). Besides using the *E. faecium* 007 strain, we performed infection assays using other clinical isolates of *E. faecium,* including the strain 1,231,410 vancomycin resistant and the strain D344R ampicillin resistant. We also employed the strain 2158 that was previously evaluated in the mouse peritonitis model [32]. We observed that these strains were also capable of infecting and killing *G. mellonella* (Fig. 1B). In addition, we verified that the virulence capability of the strain 2158 in *G. mellonella* was well correlated with the virulence profile of the same strain in the mouse peritonitis model [32].

**Figure 2:**
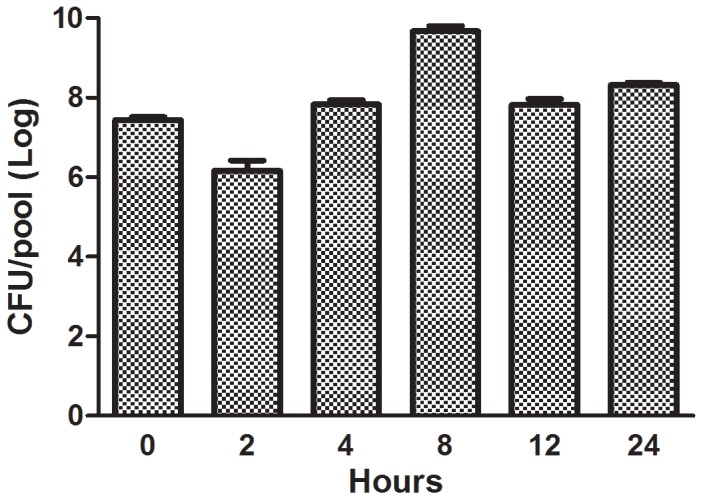
Number of bacterial cells in *G. mellonella* hemolymph post *E. faecium* E007 infection. The caterpillars were infected with 5.3×10^7^ CFU/larva and were maintained at 37°C. Number of bacterial cells was quantified from pools of five larvae hemolymph per time-point (0, 2, 4, 8, 12 and 24 h after infection). Bars represent mean and the standard deviation of three pools per time-point.

The number of bacterial cells in the hemolymph was measured at 0, 2, 4, 8, 12 and 24 h after larvae were infected with a lethal dose (5×10^7^ CFU/larva) of the *E. faecium* strain E007 (time 0 was immediately after injection). As shown in Fig. 2, during the first 2 h after injection the CFU decreased, suggesting an initially effective immune response to the infection. However, at 8 hours after the infection the maximum number of *E. faecium* was recovered (4.8×10^9^ CFU/pool).

**Figure 3:**
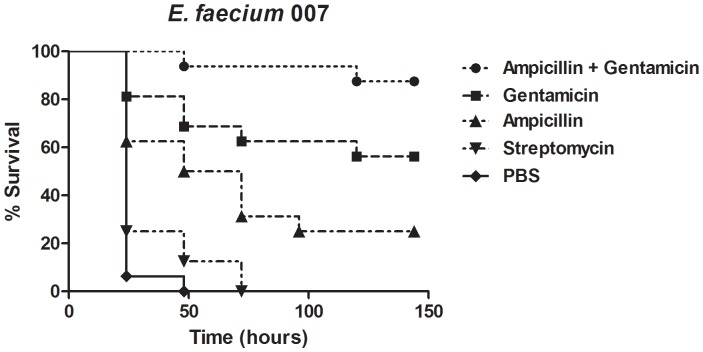
Antimicrobial drugs prolong the survival of *G. mellonella* caterpillars infected by *E. faecium.* We examined the role of the most commonly used agents (alone or in association) for enterococcal infection by administering single doses of ampicillin, gentamicin, streptomycin and the association of ampicillin and gentamicin. The antibiotics were administered within 2 h after larvae were infected with 5.6×10^7^ CFU/larva of *E. faecium* E007. A control group received the *E. faecium* E007 inoculum and PBS instead of antibiotics. Treatment with ampicillin (*P* = 0.0003), gentamicin (*P* = 0.0001) and the combination of ampicillin and gentamicin (*P* = 0.0001) significantly prolonged the survival of *G. mellonella* caterpillars when compared to control. However, streptomycin was not effective against *E. faecium* E007 (*P* = 0.0995). A representative example was used for each group.

An important question about the *G. mellonella*-*E. faecium* infection model is whether it could serve for testing antibacterial agents. We explored this possibility by assessing the efficacy of single and combinatorial antibiotic treatments for *G. mellonella* caterpillars infected by *E. faecium*. We evaluated the monotherapy with the antibiotics ampicillin (150 mg/kg), streptomycin (15 mg/kg) and gentamicin (6 mg/kg). Another experimental larvae group was administered the combination of ampicillin (150 mg/kg) and gentamicin (6 mg/kg) to evaluate the ability of the model to assess combinatorial treatment. The injection of a single dose of antibiotics ampicillin, and gentamicin prolonged the survival of *G. mellonella* caterpillars. However, for streptomycin no statistically significant difference was observed. Among the larvae that received single antibiotic, gentamicin treatment led to greater than 50% larvae survival up to 7 days after infection. Moreover, the group that received the combination treatment consisting of ampicillin and gentamicin showed the highest survival rate (more than 80% after 7 days) when compared to the other groups treated with a single antibiotic (Fig. 3). The combination of an aminoglycoside (gentamicin) with a cell-wall-active antibiotic (such as ampicillin) is the most widely antibacterial treatment for severe enterococcal infections [30].

After we verified that *G. mellonella* infected by *E. faecium* can be treated by antibacterial agents, we asked whether this host-pathogen system could be used to study aPDT. The first step was to assess potential toxic effects of PS to the larvae and whether they promoted melanization. We selected the widely used PS methylene blue (MB) for a number of reasons, including its low reported host toxicity, easy availability and broad clinical applicability [26]–[27]. Injection of larvae with MB at 1 mM did not yield melanization, death or other visible toxic effects. Additionally, the comparison of the *E. faecium* infected groups that received a second injection of MB or PBS did not show any substantial difference between the groups (data not shown).

A second preliminary step to simulate an *in vivo* aPDT study involved assessing the effects of the exposure of *G. mellonella* to red light only, before or after the infection, employing survival assays. There is evidence in other systems that red light may trigger immune responses and the absorption of red light by mitochondrial respiratory chain components may result in the increase of reactive oxygen species (ROS), and adenosinetriphosphate (ATP) or cyclic AMP, that initiate a signaling cascade, which promotes cellular proliferation and cytoprotection. Also, red light may stimulate defense cells to increase phagocytosis and to produce proteolytic enzymes [33], [34]. Groups of larvae exposed to red light alone before or after infection were compared to infected larvae that received no light exposure. No difference between the groups was observed in both experiments indicating that red light alone is not toxic to the larvae and did not alter the larvae immune response to infection (data not shown).

In order to find the optimal dose-response to MB-mediated-PDT, we evaluated 10 groups of larvae that were infected with the clinical isolate *E. faecium-*E007 and received MB injection (10 µL of 1 mM). We gradually increased the light exposure time. More specifically, 8 groups were exposed to red light at different fluences (0.9, 1.8, 3.6, 5.4, 7.2, 10.8, 14.4 and 18 J/cm^2^, corresponding to 30, 60, 120, 180, 240, 360, 480 and 600 seconds of irradiation), while two control groups received injection of PBS or MB with no light exposure. After irradiation, the survival rate of *G. mellonella* was counted 24 h post *E. faecium* infection. The best survival rate was reached with 30 seconds of irradiation (0.9 J/cm^2^). We found that after 120 seconds of light exposure that corresponded to 3.6 J/cm^2^, killing of *G. mellonella* was significantly higher compared to the control groups (P = 0.0023) indicating that the aPDT at that time exposure level was lethally toxic to the host (data not shown).

**Figure 4:**
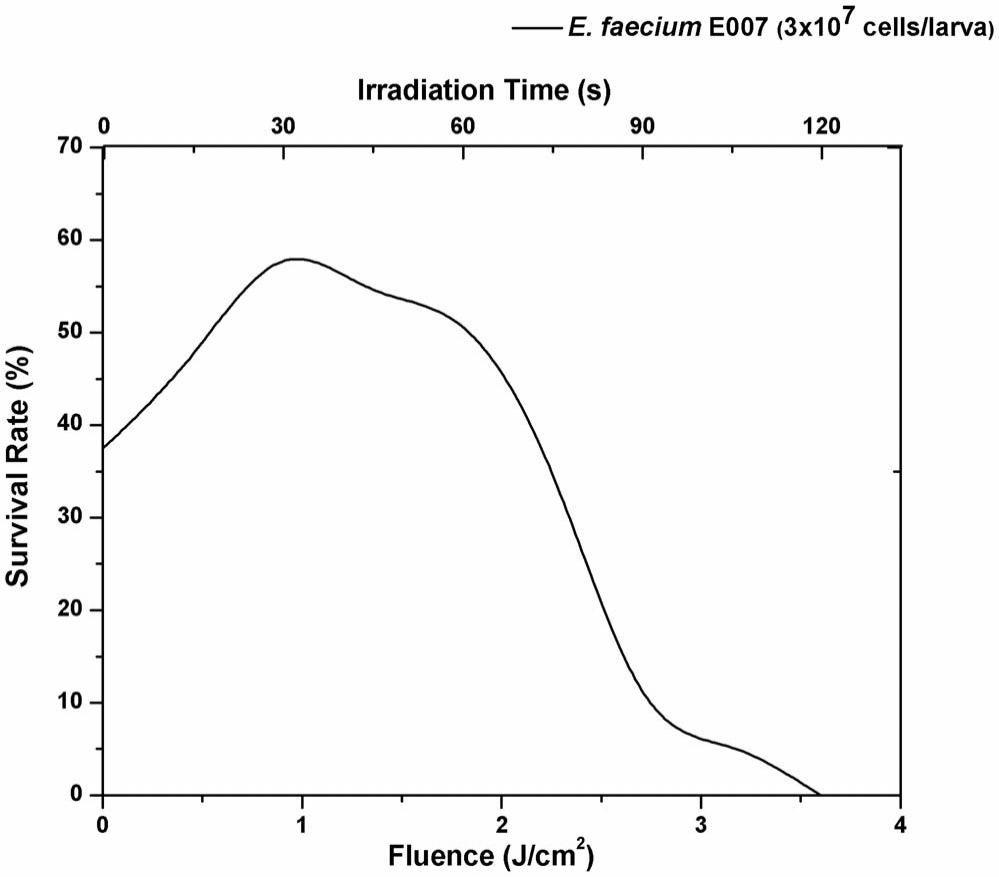
Dose-response 24 h after infected *G. mellonella* by *E. faecium* E007 were exposed to antimicrobial PDT. Larvae were infected with 3×10^7^ CFU/larva of *E. faecium* E007. Best result was found when the fluence of 0.9 J/cm^2^ was applied.

Next, a finer evaluation was performed to establish the optimum light dosimetry and 8 additional groups were divided analyzing the photodynamic effects at 15, 30, 45, 60, 75, 90, 105 and 120 seconds of irradiation (0.45, 0.9, 1.35, 1.8, 2.25, 2.7 and 3.6 J/cm2) and once again 0.9 J/cm^2^ (30 seconds of irradiation) provided the best survival rate (Fig. 4).

**Figure 5:**
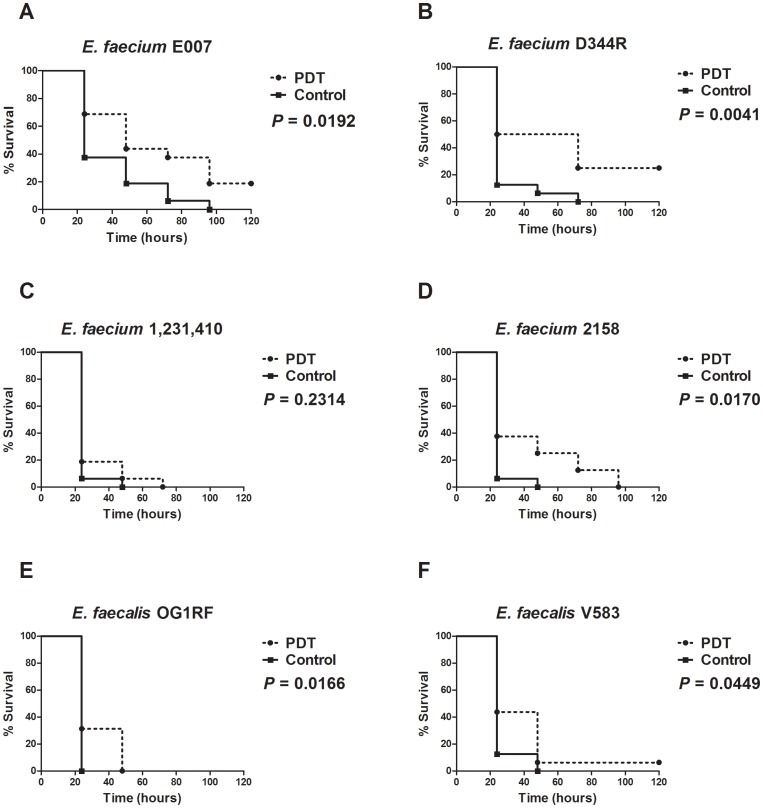
Killing of *G. mellonella* by *E. faecium* and *E. faecalis* exposed to antimicrobial PDT. In the aPDT group, the larvae received the PS injection 90 min after the bacterial infection. In order to allow a good dispersion of the PS into the insect body, we waited at least 30 additional min after the PS injection prior to the light irradiation. Control group received PS without light exposure. A) *E. faecium* E007 tetracycline resistant (3.0×10^7^ CFU/larva), B) *E. faecium* D344R ampicillin resistant (1.08×10^7^ CFU/larva), C) *E. faecium* 1,231,410 vancomycin resistant (4.8×10^7^ CFU/larva), D) *E. faecium* 2158 used in the mouse peritonitis model (1.25×10^7^ CFU/larva), E) *E. faecalis* OG1RF rifampin and fusidic acid resistant (1.7×10^6^ CFU/larva), F) *E. faecalis* V583 vancomycin resistant (1.3×10^6^ CFU/larva). A representative example was used for each group.

A further experimental procedure was designed to study the effects of aPDT, mediated by MB (1 mM) and red light at 0.9 J/cm^2^, on *Galleria* survival when infected by six different bacteria strains. We tested different strains of *E. faecium,* including *E. faecium* E007 tetracycline resistant, *E. faecium* D344R ampicillin resistant, *E. faecium* 1,231,410 vancomycin resistant, and *E. faecium* 2158 used in the mouse peritonitis model [32]. We also tested two strains of *E. faecalis: E. faecalis* OG1RF (a rifampin and fusidic acid resistant laboratory derivative of an isolate from a child with rampant caries [35] and *E. faecalis* V583, that was the first vancomycin resistant enterococcal strain isolated in the USA [36]. We observed that aPDT, prolonged significantly the larvae survival in most of the clinical isolates when compared to non-PDT treated larvae, except of the vancomycin resistant *E. faecium* 1,231,410 (Fig. 5A, B, C, D, E, F).

**Figure 6:**
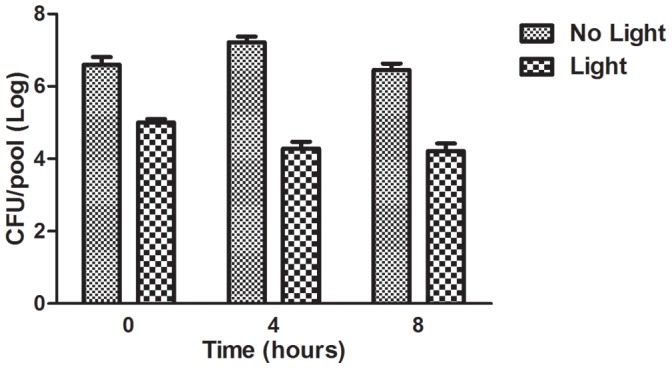
Number of bacterial cells in *G. mellonella* hemolymph over time post antimicrobial PDT treatment. Larvae were infected with 4.8×10^7^ CFU/larva of *E. faecium* 007 and were maintained at 37°C. After 90 min post-infection, the PS was injected. We waited additional 30 min prior to light irradiation. After light irradiation, the number of bacterial cells was quantified from pools of five larvae hemolymph per time point (0, 4 and 8 h after PDT that corresponded to 2, 6 and 10 h after infection). All PDT exposed groups resulted in significantly bacterial burden reduction when compared to the control group that was not exposed to PDT of each studied time-point (Student *t* test considering statistical significance with *P*<0.05: immediately *P* = 0.0080, 4 h *P* = 0.0001, 8 h *P* = 0.0010). Bars and Error bars represent respectively the mean and standard deviation of three pools per time point.

As noted on the previous section the killing of larvae depends on the number of bacteria inoculated (Fig. 1A) and the most probable explanation for the prolonged survival of the infected larvae after MB-mediated PDT is the reduction of the bacterial tissue burden. We therefore measured CFU immediately after aPDT, (time 0) as well as 4 and 8 h post-PDT treatment using larvae infected by *E. faecium* 007. We compared the hemolymph burden of aPDT-treated larvae with non-treated larvae. The aPDT effect that reduces bacterial cell viability, would occur immediately upon light exposure, as the singlet oxygen (the main PDT pathway that promotes cell death) lifetime in biological systems has been reported to be shorter than 0.04 µs [37]. A significant reduction in the CFU number was also observed at 4 and 8 h post-PDT treatment (Fig. 6). Even though there was still a significant bacterial burden, it is reasonable to assume that enterococci were impaired by the non-lethal oxidative damage which may make them more susceptible to insect immunity, resulting in a greater reduction in bacterial burden (4 and 8 h after PDT compared to time 0) and therefore prolonged the survival of PDT exposed hosts.

**Figure 7:**
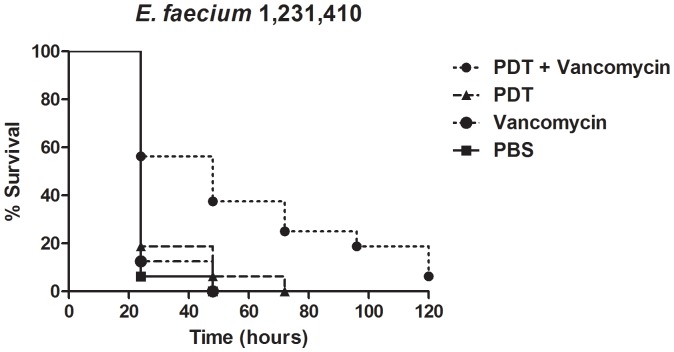
Killing of *G. mellonella* caterpillars after infection by VRE *E. faecium* 1,231,410, treated by administration of vancomycin (50 mg/kg), antimicrobial PDT, both in a combined therapy, or PBS (Control). The caterpillars received injection of 1.3×10^7^ CFU/larva and were maintained at 37°C. The combined treatment with aPDT followed by vancomycin injection resulted in significantly lower death rate when compared to treatment with PBS (*P* = 0.0012), vancomycin only (*P* = 0.0025) or aPDT alone (*P* = 0.0095). A representative example was used for each group.

We also evaluated the hypothesis that aPDT might permeabilize the microbial cell wall making vancomycin-resistant enterococci susceptible to vancomycin. Therefore, we employed the *G.mellonella-E. faecium* developed system to assess the sequential applicationof aPDT with antibiotics (Fig. 7). Larvae infected by a VRE strain were treated with MB-mediated PDT or with vancomycin. Neither therapy alone significantly prolonged larvae survival. However the sequential challenge employing aPDT followed by vancomycin led to a remarkable increase in the survival of caterpillars. The survival of *G. mellonella* infected by *E. faecium* 1,231,410, a vancomycin resistant clinical isolate, was more pronounced with a sequential treatment employing MB-mediated PDT followed by a single dose of vancomycin when compared to infected caterpillars treated with PDT alone or subjected to one dose of vancomycin.

## Discussion

In this report, we describe the use of *G. mellonella* larvae to develop an invertebrate host model system for evaluation of a variety of antimicrobial treatments against *E. faecium*, including aPDT and antibiotics. First, we performed a set of experiments to elucidate the *G. melllonella* host response following *E. faecium* infection. We found that killing of *G. mellonella* larvae depended on the number of bacteria inoculated, and all *E. faecium* strains tested were capable of infecting and killing *G. mellonella.* In addition, treatment with clinically approved antibiotics prolonged caterpillar survival infected by *E. faecium.* Then we utilized this model in order to outline the first invertebrate model for the study of aPDT and demonstrated that aPDT results in a significant reduction in the CFU number immediately upon light exposure as well as 4 and 8 h post-PDT treatment.

*G. mellonella* has been used to study the host-pathogen interaction as an alternative host model to mammalian hosts [29]–[31], [39]–[51]. As variation of the initial bacterial inoculum can considerably affect the *G. mellonella* infection, we injected 10^5^, 10^6^ and 10^7^ CFU/larva of *E. faecium* E007 in *G. mellonella* resulting in 20, 60 and 100% of mortality, respectively, after 72 h of infection. Similar mortality patterns were observed in studies employing the opportunistic pathogens *S. aureus* and *E. faecalis*. Peleg et al. [52] found mortality rates of 98 and 100% after 72 h of infection with 10^6^ and 10^7^ CFU/larva. Gaspar et al. [53] demonstrated that *E. faecalis* strains were able to kill between 60 and 98% of *G. mellonella* larvae with inocula about 2×10^6^ CFU/larva (48 h post-infection).

In this study we verified that a set of *E. faecium* multidrug resistant clinical isolates was capable to infect and kill *G. mellonella.* In a recent study, Lebreton et al. [40] showed that *G. mellonella* larvae were susceptible to infection by a variety of *E. faecium* hospital-adapted, commensal or animal isolates as well as mutant strains with deletion of virulence genes. The authors suggested that *G. mellonella* could be a suitable and convenient surrogate model to study *E. faecium* susceptibility to host defenses and the role of suspected virulence factors in the colonization process. However, the *E. faecium* strains evaluated by Lebreton et al. [40] exhibited reduced pathogenicity for *G. mellonella* compared to the results obtained in the present study. Interestingly, the vancomycin resistant strain 1,231,410 which was evaluated in both studies showed in our experiment setting a mortality rate of 100% after 50 h of injection with 4.8×10^7^ CFU/larva. Lebreton et al. [40] reported approximately 10% of mortality 50 h post infection by *E. faecium* 1,231,410 (2×10^6^ CFU/larva). Besides the inoculum concentration, the differences between our results and the data obtained by Lebreton et al. [40] can be explained by the *G. mellonella* lineage. We used *G. mellonella* within 7 days from shipment without a food source while Lebreton et al. [40] used larvae starved for 24 h. Recently, Banville et al. [54] demonstrated that the deprivation of *G. mellonella* larvae of food leads to a reduction in cellular immune responses and an increased susceptibility to infection.

*G. mellonella* can be treated by administration of traditional antimicrobial agents [38]. Treatment efficacy of Gram-positive bacterial infection with clinically approved antibiotics was recently reported in this model by Desbois et al. [39]. We observed that the injection of a single dose of the antibiotics ampicillin, and gentamicin prolonged the survival of *G. mellonella* caterpillars infected by *E. faecium*. The combination of an aminoglycoside (gentamicin) with a cell-wall-active antibiotic (such as ampicillin) is the most widely used antibacterial treatment for severe enterococcal infections [55]. We also found a better result using the combination of ampicillin and gentamicin (more than 80% survival rate after 7 days).

The emergence of multidrug resistance (MDR) involves a variety of pathogenic microorganisms and antimicrobial agents. As a consequence MDR has prompted the investigation and development of new and alternative antimicrobial technologies and countermeasures, of which aPDT has emerged as an effective approach to selective destruction of pathogens [24], [56]–[58]. *In vivo* antimicrobial PDT studies have been performed in vertebrate models, such as mice [59]–[61]. However, the high cost, together with the laborious and time consuming nature of the work may limit the number of variables studied, as well as the number of strains or species tested in a same experiment. *G. mellonella* as a model to study *in vivo* antimicrobial PDT can be very useful, especially when studying with different phenotypic features or different species of pathogen.

To the best of our knowledge, this is the first time an insect model host has been used to study antimicrobial PDT. In order to evaluate the *G. mellonella* system as a model for antimicrobial PDT, a preliminary set of experiments was performed with different groups of larvae that each received different PDT doses. Usually, a higher dose of PDT would be expected to provide better results in bacterial number reduction, but when applied in this insect model host, it was found that high-dose PDT had no effect on prolonging the survival rate when compared to non-exposed larvae. The working hypothesis is that higher PDT doses could promote damage in host tissues or on the host immune response. When a low dose of PDT was selected for application it was potent in the microorganisms and could be tolerated by *G. mellonella* larva without toxicity. Low doses of PDT can be also efficient, especially, in Gram-positive bacteria due their permeable cell wall.

In order to avoid host damage we applied a low antimicrobial dose therefore we found only a modest bacterial cell burden reduction. It is plausible that this sub-lethal PDT dose promotes bacterial cell-wall damage, thus facilitating the insect immune system response to clear the infection. With a weaker or permeable cell wall, bacteria could become easily phagocytized by *G. mellonella* hemocytes, and/or more susceptible to humoral insect immune response, by antimicrobial peptide action. This could explain the significant caterpillar survival rate in PDT exposed groups. The analysis implies that the precise mechanistic aspects of the pathogen photoinactivation in the caterpillar remain elusive. The same reservations applies in many occasions for the *in vitro* PDI explorations [62]. A comprehensive experimental design with emphasis in assessing the killing rate and the cell wall damage following *in vitro* exposure of *E. faecium* to different light levels will be essential to dissect the mechanism of the selective *E. faecium* photoinactivation in the host.

It has been demonstrated that photodynamic inactivation affect fungal cell wall and subsequently enhances the efficacy of antifungals [24]. This prompt the formulation of the hypothesis that the low PDT dose could also affect the bacteria cell wall. If the hypothesis holds truth it will be safe to assume, that the sequential application of aPDT and antimicrobial compounds such as antibiotics could act synergistically in treating the infection. It is known that vancomycin resistance by enterococci is considered the paradigm of the post-antibiotic era [13]. Conventional antimicrobial therapy could be combined with aPDT as an adjunct therapy [58]. The combination of PDT with antimicrobials has been used with success when compared to either approach [23], [63].

Importantly, we observed that the *G. mellonella* larvae survival after infection by a VRE strain was prolonged when vancomycin was administered after aPDT. When vancomycin or aPDT were applied alone no extension of caterpillar survival was observed. It is entirely possible that the permeabilization of the bacterial cell wall by the sub-lethal aPDT dose, makes it more susceptible to vancomycin. The exact mechanism by which aPDT makes VRE susceptible to vancomycin remains to be clarified. Again, further experimentation will be required to address the exact mechanism of this promising therapeutic modality for VRE infections or other resistant pathogens.

Overall, the first facile, whole animal alternative model host for aPDT testing is described. This invertebrate animal model provides a novel valuable tool to explore combinatorial aPDT-based treatments. It is logical to anticipate that the model described will be used to study the *in vivo* efficacy of new photosensitizers, and PDT-based protocols, without the ethical, financial and logistical barriers of mammalian models.

## References

[1] Sifri CD, Mylonakis E, Singh KV, Qin X, Garsin DA, et al (2002) Virulence effect of *Enterococcus faecalis* protease genes and the quorum-sensing locus fsr in *Caenorhabditis elegans* and mice. Infect Immun 70: 5647–5650. 12228293 10.1128/IAI.70.10.5647-5650.2002 PMC128331

[2] Bhardwaj A, Kapila S, Mani J, Malik RK (2009) Comparison of susceptibility to opsonic killing by in vitro human immune response of Enterococcus strains isolated from dairy products, clinical samples and probiotic preparation. Int J Food Microbiol 128: 513–515. 19004514 10.1016/j.ijfoodmicro.2008.10.010

[3] Michaux C, Sanguinetti M, Reffuveille F, Auffray Y, Posteraro B, et al (2011) SlyA is a transcriptional regulator involved in the virulence of *Enterococcus faecalis* . Infect Immun 79: 2638–2645. 21536798 10.1128/IAI.01132-10 PMC3191995

[4] de Regt MJ, Willems RJ, Hene RJ, Siersema PD, Verhaar HJ, et al (2010) Effects of probiotics on acquisition and spread of multiresistant enterococci. Antimicrob Agents Chemother 54: 2801–2805. 20404120 10.1128/AAC.01765-09 PMC2897316

[5] Kristich CJ, Little JL, Hall CL, Hoff JS (2011) Reciprocal regulation of cephalosporin resistance in *Enterococcus faecalis* . MBio 2: e00199–00111. 22045988 10.1128/mBio.00199-11 PMC3202758

[6] Maadani A, Fox KA, Mylonakis E, Garsin DA (2007) *Enterococcus faecalis* mutations affecting virulence in the *Caenorhabditis elegans* model host. Infect Immun 75: 2634–2637. 17307944 10.1128/IAI.01372-06 PMC1865755

[7] Paganelli FL, Willems RJ, Leavis HL (2012) Optimizing future treatment of enterococcal infections: attacking the biofilm? Trends Microbiol 20: 40–49. 22169461 10.1016/j.tim.2011.11.001

[8] Lindenstrauss AG, Pavlovic M, Bringmann A, Behr J, Ehrmann MA, et al (2011) Comparison of genotypic and phenotypic cluster analyses of virulence determinants and possible role of CRISPR elements towards their incidence in *Enterococcus faecalis* and *Enterococcus faecium* . Syst Appl Microbiol 34: 553–560. 21943678 10.1016/j.syapm.2011.05.002

[9] Moy TI, Mylonakis E, Calderwood SB, Ausubel FM (2004) Cytotoxicity of hydrogen peroxide produced by *Enterococcus faecium* . Infect Immun 72: 4512–4520. 15271910 10.1128/IAI.72.8.4512-4520.2004 PMC470665

[10] Nandakumar R, Madayiputhiya N, Fouad AF (2009) Proteomic analysis of endodontic infections by liquid chromatography-tandem mass spectrometry. Oral Microbiol Immunol 24: 347–352. 19572900 10.1111/j.1399-302X.2009.00520.x PMC2744886

[11] Zhu X, Wang Q, Zhang C, Cheung GS, Shen Y (2010) Prevalence, phenotype, and genotype of *Enterococcus faecalis* isolated from saliva and root canals in patients with persistent apical periodontitis. J Endod 36: 1950–1955. 21092811 10.1016/j.joen.2010.08.053

[12] Dahlen G, Blomqvist S, Almstahl A, Carlen A (2012) Virulence factors and antibiotic susceptibility in enterococci isolated from oral mucosal and deep infections. J Oral Microbiol 4. 10.3402/jom.v4i0.10855 PMC3285953 22368771

[13] Willems RJ, Hanage WP, Bessen DE, Feil EJ (2011) Population biology of Gram-positive pathogens: high-risk clones for dissemination of antibiotic resistance. FEMS Microbiol Rev 35: 872–900. 21658083 10.1111/j.1574-6976.2011.00284.x PMC3242168

[14] Maisch T, Hackbarth S, Regensburger J, Felgentrager A, Baumler W, et al (2011) Photodynamic inactivation of multi-resistant bacteria (PIB) - a new approach to treat superficial infections in the 21st century. J Dtsch Dermatol Ges 9: 360–366. 21114627 10.1111/j.1610-0387.2010.07577.x

[15] Rice LB (2008) Federal funding for the study of antimicrobial resistance in nosocomial pathogens: no ESKAPE. J Infect Dis 197: 1079–1081. 18419525 10.1086/533452

[16] Hamblin M, Hasan T (2004) Photodynamic therapy: a new antimicrobial approach to infectious disease? Photochem Photobiol Sci 3: 436–450. 15122361 10.1039/b311900a PMC3071049

[17] Wainwright M, Byrne MN, Gattrell MA (2006) Phenothiazinium-based photobactericidal materials. J Photochem Photobiol B 84: 227–230. 16713280 10.1016/j.jphotobiol.2006.03.002

[18] Huang L, Huang YY, Mroz P, Tegos GP, Zhiyentayev T, et al (2010) Stable synthetic cationic bacteriochlorins as selective antimicrobial photosensitizers. Antimicrob Agents Chemother 54: 3834–3841. 20625146 10.1128/AAC.00125-10 PMC2934952

[19] Castano A, Demidova TN, Hamblin MR (2004) Mechanisms in photodynamic therapy: part one-photosensitizers, photochemistry and cellular localization. Photodiagn Photodynam Ther 1: 279–293. 10.1016/S1572-1000(05)00007-4 PMC4108220 25048432

[20] Junqueira JC, Jorge AO, Barbosa JO, Rossoni RD, Vilela SF, et al. (2012) Photodynamic inactivation of biofilms formed by Candida spp., *Trichosporon mucoides*, and *Kodamaea ohmeri* by cationic nanoemulsion of zinc 2,9,16,23-tetrakis(phenylthio)-29H, 31H-phthalocyanine (ZnPc). Lasers Med Sci. 10.1007/s10103-012-1050-2 22278349

[21] Mitton D, Ackroyd R (2008) A brief overview of photodynamic therapy in Europe. Photodiagnosis Photodyn Ther 5: 103–111. 19356640 10.1016/j.pdpdt.2008.04.004

[22] St. Denis T, Dai T, Izikson A, Astrakas C, Anderson RR, et al (2011) All you need is light. Antimicrobial photoinactivation as an evolving and emerging discovery strategy against infectious disease Virulence 2: 1–12. 10.4161/viru.2.6.17889 PMC3260545 21971183

[23] Di Poto A, Sbarra MS, Provenza G, Visai L, Speziale P (2009) The effect of photodynamic treatment combined with antibiotic action or host defence mechanisms on *Staphylococcus aureus* biofilms. Biomaterials 30: 3158–3166. 19329182 10.1016/j.biomaterials.2009.02.038

[24] Fuchs BB, Tegos GP, Hamblin MR, Mylonakis E (2007) Susceptibility of *Cryptococcus neoformans* to photodynamic inactivation is associated with cell wall integrity. Antimicrob Agents Chemother 51: 2929–2936. 17548495 10.1128/AAC.00121-07 PMC1932496

[25] Kharkwal GB, Sharma SK, Huang YY, Dai T, Hamblin MR (2011) Photodynamic therapy for infections: clinical applications. Lasers Surg Med 43: 755–767. 22057503 10.1002/lsm.21080 PMC3449167

[26] Wainwright M, Phoenix DA, Rice L, Burrow SM, Waring J (1997) Increased cytotoxicity and phototoxicity in the methylene blue series via chromophore methylation. J Photochem Photobiol B 40: 233–239. 9372612 10.1016/s1011-1344(97)00061-4

[27] Pereira CA, Romeiro RL, Costa AC, Machado AK, Junqueira JC, et al (2011) Susceptibility of *Candida albicans*, *Staphylococcus aureus*, and *Streptococcus mutans* biofilms to photodynamic inactivation: an in vitro study. Lasers Med Sci 26: 341–348. 21069408 10.1007/s10103-010-0852-3

[28] Kavanagh K, Reeves EP (2004) Exploiting the potential of insects for in vivo pathogenicity testing of microbial pathogens. FEMS Microbiol Rev 28: 101–112. 14975532 10.1016/j.femsre.2003.09.002

[29] Aperis G, Fuchs BB, Anderson CA, Warner JE, Calderwood SB, et al (2007) *Galleria mellonella* as a model host to study infection by the *Francisella tularensis* live vaccine strain. Microbes Infect 9: 729–734. 17400503 10.1016/j.micinf.2007.02.016 PMC1974785

[30] Peleg AY, Jara S, Monga D, Eliopoulos GM, Moellering RC Jr, et al (2009) *Galleria mellonella* as a model system to study *Acinetobacter baumannii* pathogenesis and therapeutics. Antimicrob Agents Chemother 53: 2605–2609. 19332683 10.1128/AAC.01533-08 PMC2687231

[31] Junqueira JC, Fuchs BB, Muhammed M, Coleman JJ, Suleiman JAH, et al (2011) Oral *Candida albicans* isolates from HIV-positive individuals have similar *in vitro* biofilm-forming ability and pathogenicity as invasive *Candida* isolates. BMC Microbiology 11: 247. 22053894 10.1186/1471-2180-11-247 PMC3217868

[32] Panesso D, Montealegre MC, Rincon S, Mojica MF, Rice LB, et al (2011) The hylEfm gene in pHylEfm of *Enterococcus faecium* is not required in pathogenesis of murine peritonitis. BMC Microbiol 11: 20. 21266081 10.1186/1471-2180-11-20 PMC3039558

[33] Lins RD, Dantas EM, Lucena KC, Catao MH, Granville-Garcia AF, et al (2010) Biostimulation effects of low-power laser in the repair process. An Bras Dermatol 85: 849–855. 21308309 10.1590/s0365-05962010000600011

[34] Gao X, Xing D (2009) Molecular mechanisms of cell proliferation induced by low power laser irradiation. J Biomed Sci 16: 4. 19272168 10.1186/1423-0127-16-4 PMC2644974

[35] Dunny GM, Brown BL, Clewell DB (1978) Induced cell aggregation and mating in *Streptococcus faecalis*: evidence for a bacterial sex pheromone. Proc Natl Acad Sci U S A 75: 3479–3483. 98769 10.1073/pnas.75.7.3479 PMC392801

[36] Sahm DF, Kissinger J, Gilmore MS, Murray PR, Mulder R, et al (1989) In vitro susceptibility studies of vancomycin-resistant *Enterococcus faecalis* . Antimicrob Agents Chemother 33: 1588–1591. 2554802 10.1128/aac.33.9.1588 PMC172707

[37] Dougherty TJ, Gomer CJ, Henderson BW, Jori G, Kessel D, et al (1998) Photodynamic therapy. J Natl Cancer Inst 90: 889–905. 9637138 10.1093/jnci/90.12.889 PMC4592754

[38] Coleman JJ, Muhammed M, Kasperkovitz PV, Vyas JM, Mylonakis E (2011) Fusarium pathogenesis investigated using *Galleria mellonella* as a heterologous host. Fungal Biol 115: 1279–1289. 22115447 10.1016/j.funbio.2011.09.005 PMC3224342

[39] Desbois AP, Coote PJ (2011) Wax moth larva (*Galleria mellonella*): an in vivo model for assessing the efficacy of antistaphylococcal agents. J Antimicrob Chemother 66: 1785–1790. 21622972 10.1093/jac/dkr198

[40] Lebreton F, Le Bras F, Reffuveille F, Ladjouzi R, Giard JC, et al (2011) *Galleria mellonella* as a model for studying *Enterococcus faecium* host persistence. J Mol Microbiol Biotechnol 21: 191–196. 22286046 10.1159/000332737

[41] Gaddy JA, Arivett BA, McConnell MJ, Lopez-Rojas R, Pachon J, et al (2012) Role of Acinetobactin-Mediated Iron Acquisition Functions in the Interaction of *Acinetobacter baumannii* Strain ATCC 19606T with Human Lung Epithelial Cells, *Galleria mellonella* Caterpillars, and Mice. Infect Immun 80: 1015–1024. 22232188 10.1128/IAI.06279-11 PMC3294665

[42] Jander G, Rahme LG, Ausubel FM (2000) Positive correlation between virulence of *Pseudomonas aeruginosa* mutants in mice and insects. J Bacteriol 182: 3843–3845. 10851003 10.1128/jb.182.13.3843-3845.2000 PMC94559

[43] Miyata S, Casey M, Frank DW, Ausubel FM, Drenkard E (2003) Use of the *Galleria mellonella* caterpillar as a model host to study the role of the type III secretion system in *Pseudomonas aeruginosa* pathogenesis. Infect Immun 71: 2404–2413. 12704110 10.1128/IAI.71.5.2404-2413.2003 PMC153283

[44] Champion OL, Cooper IA, James SL, Ford D, Karlyshev A, et al (2009) *Galleria mellonella* as an alternative infection model for *Yersinia pseudotuberculosis* . Microbiology 155: 1516–1522. 19383703 10.1099/mic.0.026823-0

[45] Abranches J, Miller JH, Martinez AR, Simpson-Haidaris PJ, Burne RA, et al (2011) The collagen-binding protein Cnm is required for *Streptococcus mutans* adherence to and intracellular invasion of human coronary artery endothelial cells. Infect Immun 79: 2277–2284. 21422186 10.1128/IAI.00767-10 PMC3125845

[46] Olsen RJ, Watkins ME, Cantu CC, Beres SB, Musser JM (2011) Virulence of serotype M3 Group A Streptococcus strains in wax worms (*Galleria mellonella* larvae). Virulence 2: 111–119. 21258213 10.4161/viru.2.2.14338 PMC3100763

[47] Yasmin A, Kenny JG, Shankar J, Darby AC, Hall N, et al (2010) Comparative genomics and transduction potential of *Enterococcus faecalis* temperate bacteriophages. J Bacteriol 192: 1122–1130. 20008075 10.1128/JB.01293-09 PMC2812964

[48] Fuchs BB, Eby J, Nobile CJ, El Khoury JB, Mitchell AP, et al (2010) Role of filamentation in *Galleria mellonella* killing by *Candida albicans* . Microbes Infect 12: 488–496. 20223293 10.1016/j.micinf.2010.03.001 PMC2883670

[49] Mylonakis E, Moreno R, El Khoury JB, Idnurm A, Heitman J, et al (2005) *Galleria mellonella* as a model system to study *Cryptococcus neoformans* pathogenesis. Infect Immun 73: 3842–3850. 15972469 10.1128/IAI.73.7.3842-3850.2005 PMC1168598

[50] Fuchs BB, Mylonakis E (2006) Using non-mammalian hosts to study fungal virulence and host defense. Curr Opin Microbiol 9: 346–351. 16814595 10.1016/j.mib.2006.06.004

[51] Desalermos A, Fuchs BB, Mylonakis E (2012) Selecting an invertebrate model host for the study of fungal pathogenesis. PLoS Pathog 8: e1002451. 22319439 10.1371/journal.ppat.1002451 PMC3271057

[52] Peleg AY, Monga D, Pillai S, Mylonakis E, Moellering RC, et al (2009) Reduced susceptibility to vancomycin influences pathogenicity in *Staphylococcus aureus* infection. J Infect Dis 199: 532–536. 19125671 10.1086/596511 PMC3750955

[53] Gaspar F, Teixeira N, Rigottier-Gois L, Marujo P, Nielsen-LeRoux C, et al (2009) Virulence of *Enterococcus faecalis* dairy strains in an insect model: the role of *fsrB* and *gelE* . Microbiology 155: 3564–3571. 19696101 10.1099/mic.0.030775-0

[54] Banville N, Browne N, Kavanagh K (2012) Effect of nutrient deprivation on the susceptibility of *Galleria mellonella* larvae to infection. Virulence 3: 497–503. 23076277 10.4161/viru.21972 PMC3524148

[55] Murray PR, Rosenthal KS, Pfaller MA (2009) Medical microbiology. Philadelphia: Mosby/Elsevier. x, 947 p.

[56] Hamblin MR, O’Donnell DA, Murthy N, Rajagopalan K, Michaud N, et al (2002) Polycationic photosensitizer conjugates: effects of chain length and Gram classification on the photodynamic inactivation of bacteria. J Antimicrob Chemother 49: 941–951. 12039886 10.1093/jac/dkf053

[57] St Denis TG, Huang L, Dai T, Hamblin MR (2011) Analysis of the bacterial heat shock response to photodynamic therapy-mediated oxidative stress. Photochem Photobiol 87: 707–713. 21261628 10.1111/j.1751-1097.2011.00902.x PMC3082629

[58] Chabrier-Rosello Y, Foster TH, Mitra S, Haidaris CG (2008) Respiratory deficiency enhances the sensitivity of the pathogenic fungus *Candida* to photodynamic treatment. Photochem Photobiol 84: 1141–1148. 18248505 10.1111/j.1751-1097.2007.00280.x

[59] Junqueira JC, Martins JS, Faria RL, Colombo CED, Jorge AOC (2009) Photodynamic therapy for the treatment of buccal candidiasis in rats. Lasers Med Sci 24: 877–884. 19408038 10.1007/s10103-009-0673-4

[60] Martins JS, Junqueira JC, Faria RL, Santiago NF, Rossoni RD, et al (2011) Antimicrobial photodynamic therapy in rat experimental candidiasis: Evaluation of pathogenicity factors of *Candida albicans* . Oral Surg Oral Med Oral Pathol Oral Radiol Endod 111: 71–77. 21176823 10.1016/j.tripleo.2010.08.012

[61] Costa ACBP, Rasteiro VMC, Hashimoto ES, Araújo CF, Pereira CA, et al (2012) Effect of erythrosine- and LED-mediated photodynamic therapy on buccal candidiasis infection of immunosuppressed mice and *Candida albicans* adherence to buccal epithelial cells. Oral Surg Oral Med Oral Pathol Oral Radiol 114: 67–74. 22727094 10.1016/j.oooo.2012.02.002

[62] Vera DMA, Haynes MH, Ball AR, Dai T, Astrakas C, et al (2012) Strategies to potentiate antimicrobial photoinactivation by overcoming resistant phenotypes. Photochem Photobiol 88: 499–511. 22242675 10.1111/j.1751-1097.2012.01087.x PMC3345078

[63] Snell SB, Foster TH, Haidaris CG (2012) Miconazole induces fungistasis and increases killing of *Candida albicans* subjected to photodynamic therapy. Photochem Photobiol 88: 596–603. 22077904 10.1111/j.1751-1097.2011.01039.x PMC3297714

[64] Garsin DA, Sifri CD, Mylonakis E, Qin X, Singh KV, et al (2001) A simple model host for identifying Gram-positive virulence factors. Proc Natl Acad Sci U S A 98: 10892–10897. 11535834 10.1073/pnas.191378698 PMC58570

[65] Rice LB, Carias LL, Rudin S, Hutton R, Marshall S, et al (2009) Role of class A penicillin-binding proteins in the expression of beta-lactam resistance in *Enterococcus faecium* . J Bacteriol 191: 3649–3656. 19304851 10.1128/JB.01834-08 PMC2681891

